# Supplementary figures and images for: Phylogenetic trees of closely related bacterial species and subspecies based on frequencies of short nucleotide sequences
Source: PLoS One. 2023 Apr 20;18(4):e0268847. doi: 10.1371/journal.pone.0268847 (PMC10118083; doi:10.1371/journal.pone.0268847)

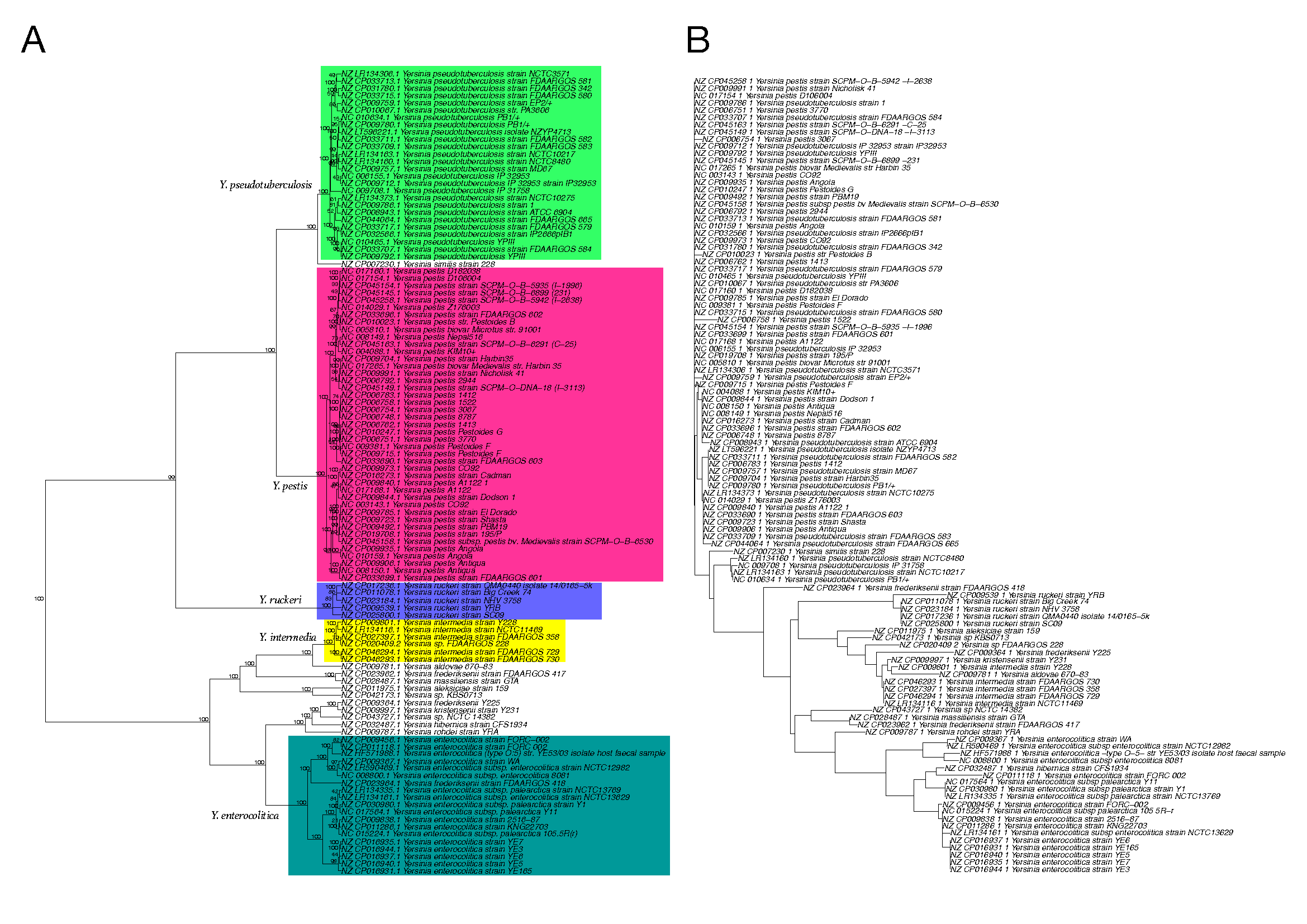

Supplement: S1 Fig — The trees were constructed using the Manhattan distance and Ward’s algorithm (A) or the neighbor-joining method (B). The numbers at the nodes indicate the percentage occurrences among 1,000 bootstrap values. Separated groups of species are highlighted. (TIFF) [file pone.0268847.s001.tiff]

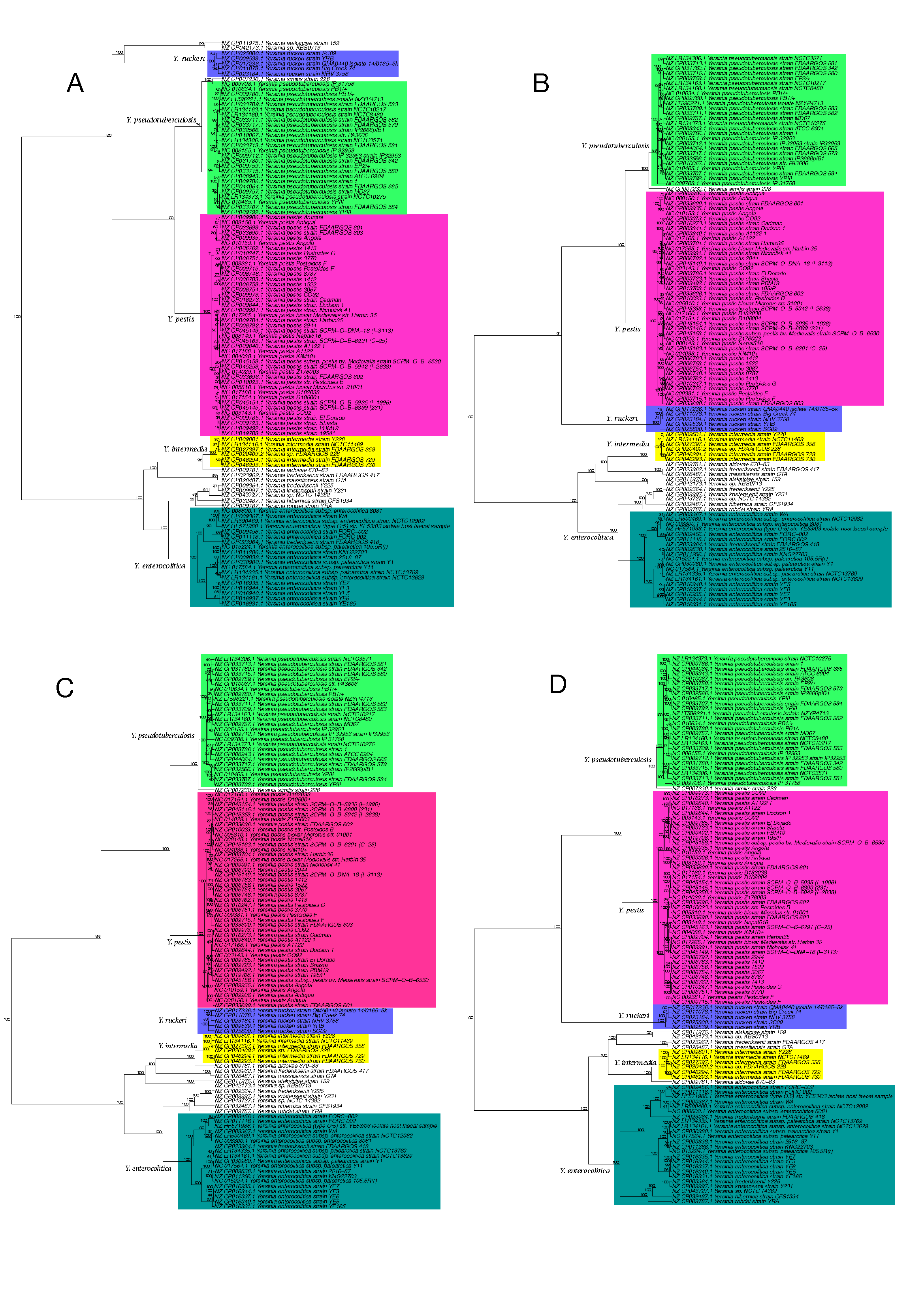

Supplement: S2 Fig — The trees were constructed using the Euclidean distance and Ward’s algorithm. The numbers at the nodes indicate the percentage occurrences among 1,000 bootstrap values. (TIFF) [file pone.0268847.s002.tiff]

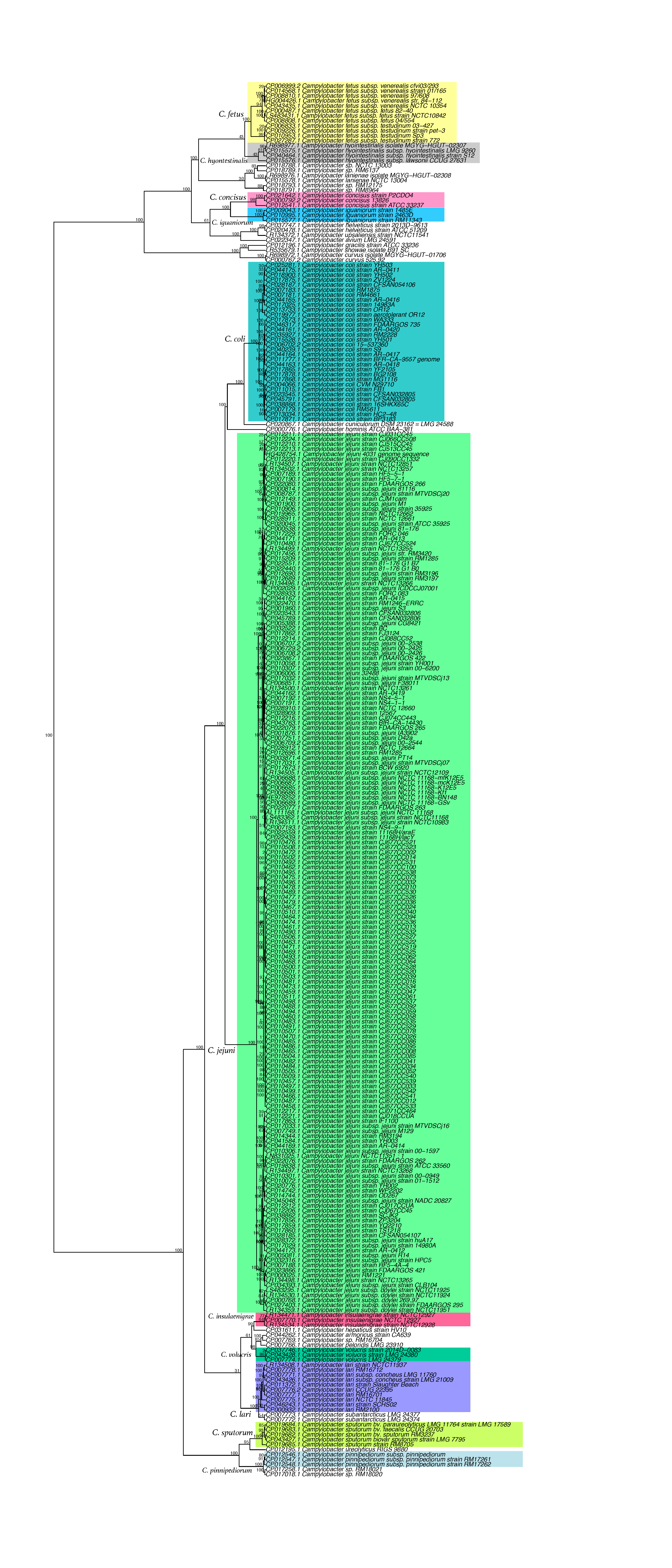

Supplement: S4 Fig — The trees were constructed using the Euclidean distance and Ward’s algorithm. The numbers at the nodes indicate the percentage occurrences among 1,000 bootstrap values. (TIFF) [file pone.0268847.s004.tiff]

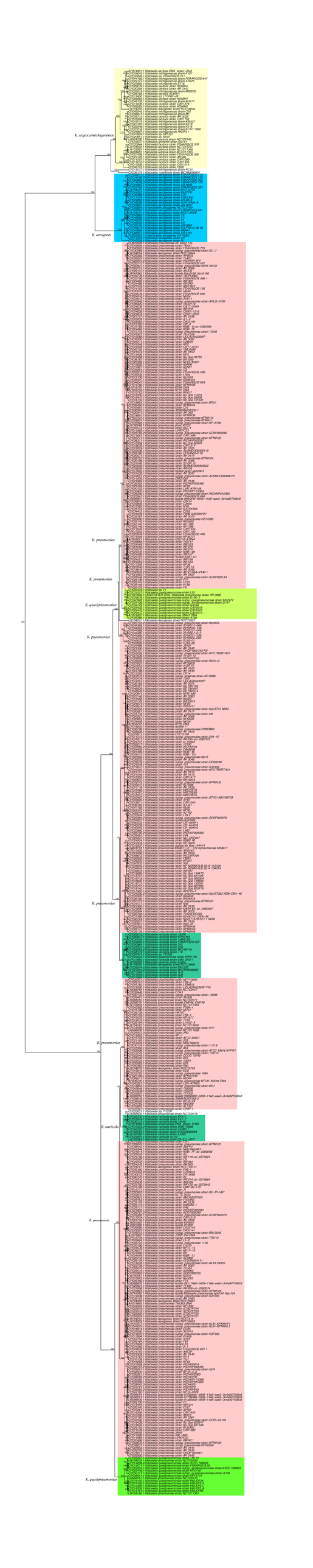

Supplement: S5 Fig — The trees were constructed using the Euclidean distance and Ward’s algorithm. The numbers at the nodes indicate the percentage occurrences among 1,000 bootstrap values. (TIFF) [file pone.0268847.s005.tiff]

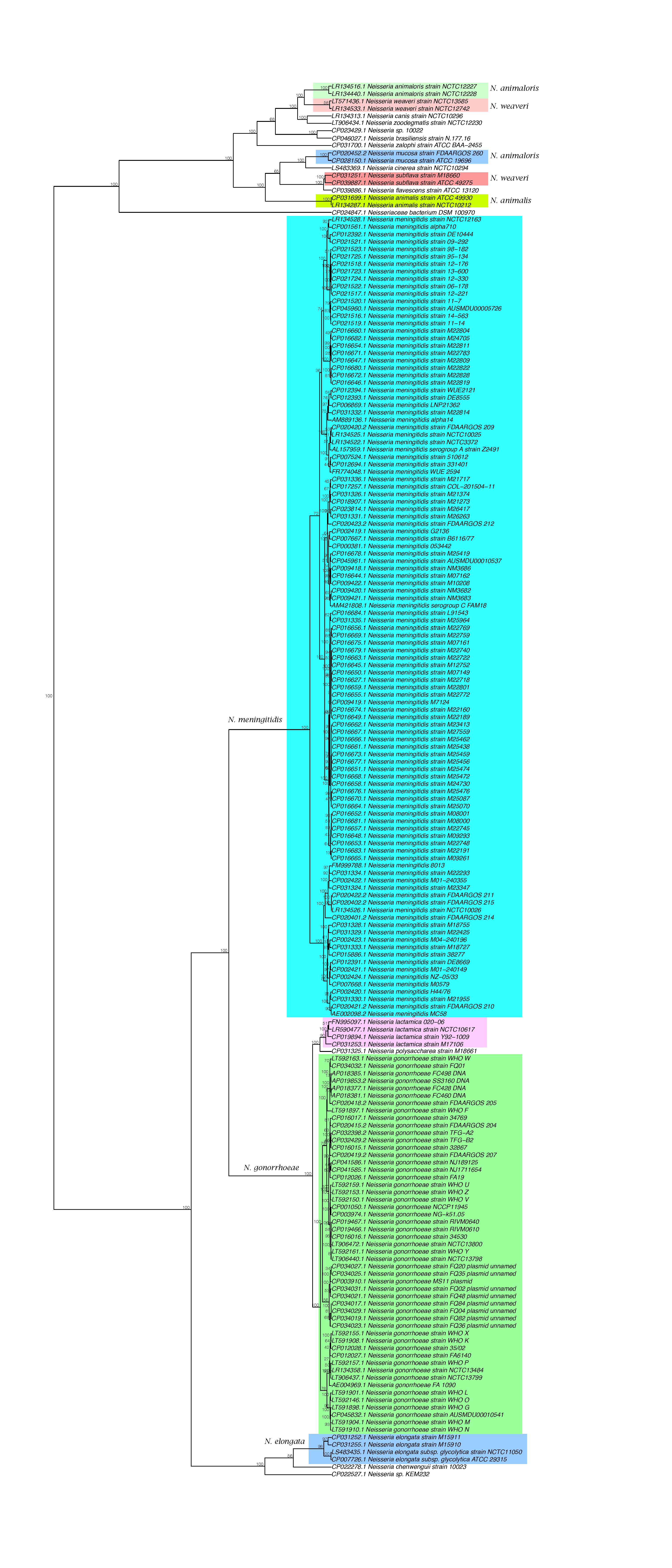

Supplement: S6 Fig — The trees were constructed using the Euclidean distance and Ward’s algorithm. The numbers at the nodes indicate the percentage occurrences among 1,000 bootstrap values. (TIFF) [file pone.0268847.s006.tiff]
